# *GGPS1* ‐associated muscular dystrophy with and without hearing loss

**DOI:** 10.1002/acn3.51633

**Published:** 2022-07-23

**Authors:** Rauan Kaiyrzhanov, Luke Perry, Clarissa Rocca, Maha S. Zaki, Heba Hosny, Cristiane Araujo Martins Moreno, Rahul Phadke, Irina Zaharieva, Clara Camelo Gontijo, Christian Beetz, Veronica Pini, Mojtaba Movahedinia, Edmar Zanoteli, Stephanie DiTroia, Sandrine Vuillaumier‐Barrot, Arnaud Isapof, Mohammad Yahya Vahidi Mehrjardi, Nasrin Ghasemi, Anna Sarkozy, Francesco Muntoni, Sandra Whalen, Barbara Vona, Henry Houlden, Reza Maroofian

**Affiliations:** ^1^ Department of Neuromuscular Disorders UCL Queen Square Institute of Neurology London WC1N 3BG UK; ^2^ The Dubowitz Neuromuscular Centre University College London, Great Ormond Street, Institute of Child Health and MRC Centre for Neuromuscular Diseases, Neurosciences Unit, Great Ormond Street Hospital London UK; ^3^ MRC UCL International Centre for Genomic Medicine in Neuromuscular Diseases (ICGNMD) London UK; ^4^ Clinical Genetics Department Human Genetics and Genome Research Division, National Research Centre 12622 Cairo Egypt; ^5^ National Institute of Neuromotor System Cairo Egypt; ^6^ Diagnostic Department Centogene GmbH 18055 Rostock Germany; ^7^ Department of Neurology School of Medicine of Universidade de Sao Paulo Sao Paulo Brazil; ^8^ Children Growth Disorder Research Center Shahid Sadoughi University of Medical Sciences Yazd Iran; ^9^ Program in Medical and Population Genetics and Center for Mendelian Genomics Broad Institute of MIT and Harvard Cambridge Massachusetts USA; ^10^ Service de Biochimie et génétique, APHP Hôpital Bichat‐Claude Bernard 75018 Paris France; ^11^ Service de neuropédiatrie APHP, Sorbonne Université, Hôpital Armand Trousseau 75012 Paris France; ^12^ Medical Genetics Research Center Shahid Sadoughi University of Medical Sciences Yazd Iran; ^13^ Abortion Research Centre Yazd Reproductive Sciences Institute, Shahid Sadoughi University of Medical Sciences 8916978477 Yazd Iran; ^14^ NIHR Great Ormond Street Hospital Biomedical Research Centre Great Ormond Street Institute of Child Health, University College London London UK; ^15^ UF de Génétique Clinique Centre de Référence Maladies Rares Anomalies du Développement et Syndromes Malformatifs, AP‐HP. Sorbonne Université, Hôpital Armand Trousseau 75012 Paris France; ^16^ Institute of Human Genetics University Medical Center Göttingen Göttingen Germany; ^17^ Institute for Auditory Neuroscience and Inner Ear Lab University Medical Center Göttingen Göttingen Germany

## Abstract

Ultra‐rare biallelic pathogenic variants in geranylgeranyl diphosphate synthase 1 (*GGPS1*) have recently been associated with muscular dystrophy/hearing loss/ovarian insufficiency syndrome. Here, we describe 11 affected individuals from four unpublished families with ultra‐rare missense variants in *GGPS1* and provide follow‐up details from a previously reported family. Our cohort replicated most of the previously described clinical features of *GGPS1* deficiency; however, hearing loss was present in only 46% of the individuals. This report consolidates the disease‐causing role of biallelic variants in *GGPS1* and demonstrates that hearing loss and ovarian insufficiency might be a variable feature of the *GGPS1*‐associated muscular dystrophy.

## Introduction

Geranylgeranyl diphosphate synthase 1 (*GGPS1*) is a member of the prenyltransferase family and encodes a protein with geranylgeranyl diphosphate (GGPP) synthase activity. The enzyme catalyzes the synthesis of GGPP from farnesyl diphosphate and isopentenyl diphosphate. GGPP is an important molecule responsible for the C20‐prenylation of proteins. Prenylation provides proteins with a hydrophobic C terminus, the consequence of which is a greatly increased capacity to interact with cellular membranes, which have a high concentration of signaling molecules.^1^, ^2^ Prenylation is particularly important for small guanosine triphosphatases (GTPases) such as the Rab family, which have crucial roles in vesicular trafficking in mammalian cells.^2^ The roles of prenylated proteins in cells are well conserved across species.^1^

While monoallelic variants in *GGPS1* have been identified as a risk factor for atypical femoral bone fractures in females exposed to bisphosphonates, ultra‐rare biallelic pathogenic missense variants in *GGPS1* have recently been associated with muscular dystrophy/hearing loss/ovarian insufficiency syndrome.^2^, ^3^ Here, we describe 11 affected individuals from four previously unreported British, Iranian, Egyptian, and Brazilian families harboring ultra‐rare missense variants in *GGPS1* and provide follow‐up clinical data from the previously reported two Pakistani cases by Tucker et al. (2020).^3^

## Subjects and Methods

Two of the novel families reported here were recruited from Iran and Egypt as part of the SYNaPS project at the Institute of Neurology, University College London (UCL). The third and fourth novel families were identified through a collaboration between Great Ormond Street Institute of Child Health (UCL) and the Center for Mendelian Genomics, Broad Institute, USA and School of Medicine of Universidade de Sao Paulo, respectively. The corresponding author was contacted from Tucker et al. (2020)^3^ to obtain follow‐up details from the reported 2 persons with homozygous *GGPS1* variants. To identify the genetic cause of the disease in the affected individuals, exome sequencing on DNA extracted from probands' leucocytes and variant filtering were performed as described (Table 1 for genetic methods). To assess *Ggps1* RNA expression in the mouse cochlear epithelium at various stages (embryonic (E) 14, and postnatal (P) 1 and P7), publicly available single cell RNA‐seq data were visualized using the gene expression analysis resource (gEAR).^7^ The publicly available Mouse Cell Atlas, generated as previously described,^8^ includes single‐cell RNA sequencing data used to visualize global expression of *Ggps1* in the female E14.5 gonad (version 2.0 with >520,000 cells from over 10 mouse tissues grouped into 95 major clusters) and neonatal skeletal muscle excised from the leg (version 1.0 with >400,000 cells from over 10 mouse tissues grouped into 104 major clusters). Clusters showing expression (red) are marked in black numbers representing the cluster code defined on the Mouse Cell Atlas.^8^ The study was approved by the ethics Institutional Review Board of UCL and additional local ethics committees of the participating centres. Informed consent was obtained from all families.

**Table 1:** Clinical features of the affected individuals with biallelic *GGPS1* variants.

	Person	Family 1	Family 2	Family 3	Family 4	Family 5 (from Tucker et al., 2020)	Foley et al. 2020 (11 persons/6 families)	Tucker et al. 2020 (4 persons/2 families)
		P1–P3	P4–P9	P10	P11	P12, P13		
Variant details	Variant type	Homozygous	Homozygous	Compound heterozygous	Homozygous	Homozygous	Homozygous/compound heterozygous	Homozygous
	Variant at the cDNA level (NM_004837.4)	c.269A > G	c.439A > G	c.196A > C and c.545 T > C	c.770 T > G	c.269A > G	(c.860A > G;865C > G), (c.127C > T; 865C > G), c.866G > A, c.854 T > G	c.782G > A, c.269A > G
	Variant at the protein level	p.(Asn90Ser)	p.(Met147Val)	p.(Ill66Leu) and p.(Leu182Pro)	p.(Phe257Cys)	p.(Asn90Ser)	[p.(Tyr259Cys); p.(Arg261Gly)], [p.(Pro15Ser); p. (Arg261Gly)], p.(Arg261His), p.(Phe257Cys)	p.(Arg261His), p.(Asn90Ser)
	Methods	Makrythanasis et al.^4^	Makrythanasis et al.^4^	Pais et al. 2022,^5^ Natera‐de Benito et al.^6^	Foley et al. 2020^2^	Tucker et al. 2020^3^	Foley et al. 2020^2^	Tucker et al. 2020^3^
	Maximum allele frequency in variant databases*	<0.000001	<0.000001	Absent	Absent	<0.000001	<0.0001 to <0.000001	<0.00001
	ACMG	Likely pathogenic PS4, PP1 (moderate), PM2, PP3	Likely pathogenic PS4, PP1 (moderate), PM2, PP3	VUS (PM2, PP3, PP4)	VUS (PM2, PP3)	Likely pathogenic PS4, PP1 (moderate), PM2, PP3	Likely Pathogenic	Likely Pathogenic
Epidemiology	Sex	3F	5 M, F	F	F	1 M, 1F	F ‐ 6; M ‐ 5	F ‐3, M ‐ 1
	Consanguinity	3+	6+	−	+	2+	1 family‐no, 5 families ‐NA	NA
	Current age	11 y.o. (P1), 11 m.o. (Deceased) (P2), 8 y.o. (P3)	23.5 y.o., 4 y 8 m.o., 5 y 7 m.o., 4 y.o., 5 y.o., 30 y.o. (Deceased)	8 y.o	12 y	20 y.o., 8.5 y.o.	31, 29,22,46,45,44,14,21,22,11,8 (y.o.)	36,39,7 (y.o.)
	Age of death	P2–11 m.o.	30 y.o. (P9)	Alive	Alive	2 Alive	NA	Alive
Medical history	Type of progression	Slow	6 Slow	Slow	Slow	Moderate** (P12), Slow** (P13)	Slow	Slow (1)
	Failure to thrive	2+, 1NA(P2)	6−	−	−	−** (P12), +** (P13)	8+	NA
	Sensorineural hearing loss	3+	6−	+	+	−** (P12), +(P13)	10+, 1−	3+, 1−
	Progressive muscle weakness, onset age	1.5 y.o. (P1), NA (P2), 1.5 y.o (P3)	4 y.o., 2 y.o., 1.2 y.o, 1.5 y.o, 2 y.o., 3 y.o.	7 m.o	18 m	11 y.o.** (P12), −** (P13)	4+, less severe weakness 7	Mild (3), Severe (1)
	Joint contractures	2+, 1NA (P2)	2+, 4−	−	+	+** (2)	4+	NA
	Respiratory insufficiency, age of onset	+13 m.o. (P1) +8 m.o. (P2) +, 15 m.o. (P3)	2+ (20 y.o.−P9), 4−	+7 m.o	+10 y.o.	+,13 y.o. (P12), − (P13)	8+, 1NA	1+
	Non‐invasive ventilation	3+	1+, 5−	+	−	+ (P12), − (P13)	4+	2+
	POI	3NA	2 na 1−	NA	+	na (P12), −(P13)	3+, 3 uncertain to age	2+
	Cardiac involvement	2−, 1NA(P2)	6−	−	−	−** (2)	−	NA
	Loss of ambulation (age)	7 y.o. (P1, P3), NA(P2)	2+ (18 y.o. and 17 y.o.) 4−	−	9 y.o.	11 y.o.* (P12), −*(P13)	5+, 11 yo, 13 yo, 15,12,11	NA
Development	Age of sitting	1 y.o.(P1), NA (P1, P3)	1 y.o. (P4, P5) 8 m.o. (P6–P8), 9 m.o. (P9)	11 m.o	8 m	NA(P12), 9 m.o.*(P13)	NA	NA
	Age of walking	2.5 y.o. (P1, P3), NA(P2)	2.2 y.o., 1.7 y.o, 2 y.o., 1.4 y.o., 2.1, 2 y.o.	2 y.o	18 m	24 m.o.**, 18 m.o.**	18 m.o.	NA
	Age of first words	8 m.o. (P1, P3), 9 m.o.(P2)	8 m.o., 9 m.o. (P5, P6, P8, P9), 1 y.o		18 m	Normal (P12), Few words* (P13)	NA	NA
Physical examination	Age at last examination	11 y.o. (P1), 9 y.o. (P3), NA (P2)	23.5 y.o., 4.6 y.o, 5.5 y.o, 4 y.o., 5 y.o., 29 y.o.	7 y.o	12 y	20 y.o., 8 y.o.	28, 26 m.o.	NA
	Progressive scoliosis	−(P1), NA(P2), +(P3)	3+, 3−	+	+	+**, NA (P13)	8+	NA
	Short stature	2+, NA(P2)	4+, 2−	(25th centile for age)	−	+** (2)	NA	NA
Neurological examination	Hypotonia	3−	6+	+	+	+** (2)	NA	NA
	Muscle weakness	2+, NA(P2)	6+	+	+	+ (2)	+	2+
	Pattern of muscular weakness	3NA	Generalized (2), Proximal (4)	4 limb Proximal/Axial	Axial and proximal	LL > UL** (2)	NA	NA
	Muscle hypertrophy	2−, NA(P2)	4+, 2−	−	−	−** (2)	NA	NA
	Peripheral neuropathy	3−	6−	−	NA	−**, NA (P13)	NA	NA
	DTRs	0(P1, P3), NA(P2)	0 (2), ↓ (4)	↓	↓	↓↓** (2)	NA	NA
	Muscular atrophy	2+, NA(P2)	6+	+	+	+** (2)	NA	NA
	Myalgia	2−, NA(P2)	6+	−	NA	NA (P12), −**	NA	2+
	Muscle stiffness	2+, NA(P2)	2+, 4−	−	NA	+** (2)	NA	NA
	Gait	Broad‐based (P1, P3), NA(P2)	Non‐ambulant (2), Waddling gait (3), Normal gait (1)	Unsteady	Non‐ambulant	NA (P12), Waddling gait*	Non‐ambulant (5), NA (6)	NA
Details on Hearing loss	The age of onset	11 m.o.(P1), 6 m.o. (P2, P3)	6 Intact	Normal new‐born hearing screen. Hearing loss detected at 4 years	Childhood	Intact (P12), From birth (P13)	From neonatal to childhood	3+, Childhood
	The type of hearing loss	3 SNHL	6−	SNHL	SNHL	−(P12), SNHL (P13)	SNHL	SNHL (3)
	Laterality	Bilateral (P1, P3), NA(P2)	6−	Bilateral	Bilateral	Bilateral (P13)	Bilateral	Bilateral (3)
	Degree	Severe (P1, P3), NA(P2)	6−	Severe	Severe	80 dB ** (P13)	NA	NA
Investigations	Elevated CK (age)	3 NA	6+	+(7 m.o 1594 U/L, 4 y.o 5490 U/L)	+19 m	2+**	9+	NA
	FSH	3 NA	na (5), NA (1)	NA	High	2 NA	88.2 IU/l, 50.3 IU/l, 53.2 IU/l	60 IU/I, 35.8 IU/I
	EMG	3 Normal	Myopathic changes (6)	Myopathic	NA	2 Normal**	NA	NA
	Muscle biopsy/ histochemistry	3 NA	6 NA	(8 m.o) Type 1 fiber predominance, central nuclei, Z line streaming, mini‐cores.	Fatty infiltration and mitochondrial changes	2 Dystrophic pattern **	9+, dystrophic, with evidence of degeneration and regeneration and internalized nuclei.	NA
	Muscle MRI	3 NA	6 NA	(8 m.o) generalized muscle atrophy without fatty infiltration.	NA	2 NA	3+, fatty infiltration consistent with an underlying muscular dystrophy.	NA

P, person; NA, not available; na, not applicable; y.o., years old; m.o., months old; SNHL, sensorineural hearing loss; LL, lower limbs; UL, upper limbs; POI, primary ovarian insufficiency; FSG, follicle‐stimulating hormone; EMG, electromyography; MRI, magnetic resonance tomography; m, male; f, female; CK, creatine kinase; DTRs, deep tendon reflexes; dB, decibel.

* Database checked include: GnomAD v3, gnomAD v2.1.1, TopMED Bravo, UKBiobank, Iranome, GME Variome, In‐house Database. The total number of alleles considered was ~1,314,000.

** New information that was not previously reported by Tucker et al. (2020).

## Results

**Figure 1:**
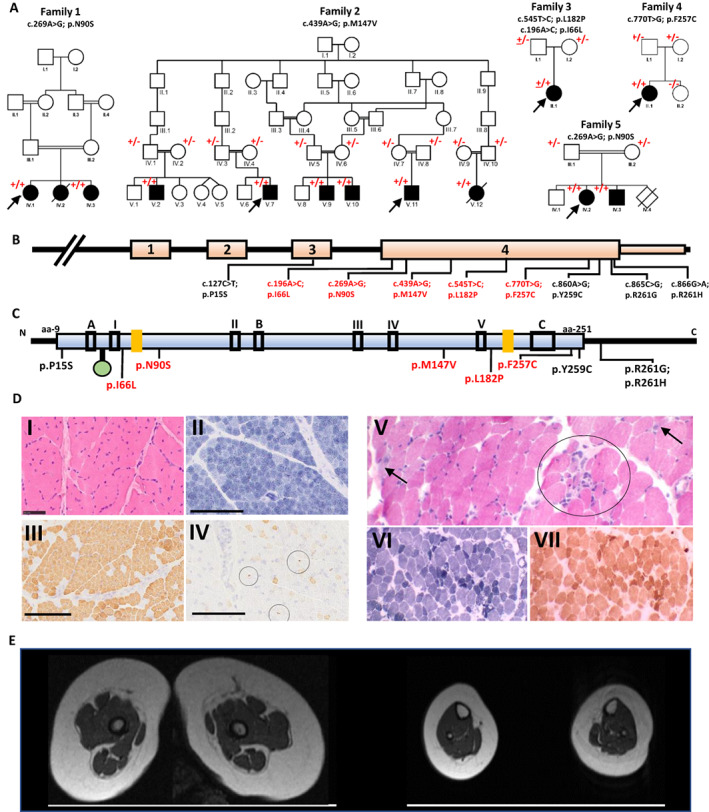
Genetic summary, muscle MRI, and muscle histopathology of the affected individuals with biallelic *GGPS1* variants. (A) Pedigrees of the five families described in the present report. Square = male; circle = female; black filled symbol = affected individual; white symbols = unaffected individuals; diagonal line = deceased individual. Double lines indicate consanguinity. The allele with the variant is indicated by the + sign. Wild type allele is indicated by the—sign. Inheritance of the compound heterozygous variant in Family 3 is indicated by the underlined + sign. (B) Schematic representation of the *GGPS1* gene showing the position of the variants. (C) Schematic diagram indicating the predicted domains and active sites of the GGPS1 protein containing 300 amino acid residues. The blue box represents the polyprenyl synthetase domain. The green circle indicates a magnesium D metal ion binding. Black boxes II, III, and V represent conserved regions that are believed to be involved in ligating the magnesium and the pyrophosphate on the allylic substrate. Box I contains two basic residues involved in IPP pyrophosphate binding. Box IV is the KT sequence containing Thr‐152 proposed to be involved in catalysis by stabilizing a carbocation intermediate. Boxes A, B, C are regions thought to be responsible for the hexameric organization of protein.^12^ The two yellow boxes are proposed active sites.^13^ (D) Muscle biopsies from affected individuals showed myopathic to dystrophic changes. Family 3 (P10): Quadriceps biopsy performed at 8 months of age (I‐IV). Hematoxylin & eosin‐stained section shows well‐populated fascicles with mild variation in fiber size (I). Staining with NADH‐TR shows normal internal architecture and predominance of type I fibers (II). Myosin heavy chain immunohistochemistry shows corresponding predominance of fibers expressing slow myosin heavy chain (III). Aberrant foetal myosin heavy chain expression of varying intensities can be seen in several scattered mature fibers, as well as a population of very small fibers (IV, circles), dispersed across the fascicles. Family 4 (P11): Biceps brachii biopsy performed at 4 years of age (V–VII). Hematoxylin and eosin‐stained section shows moderate variation in fiber size (V). There are several scattered (arrow) and clustered (circle) basophilic regenerating fibers (V). Few fibers contain internal nuclei. NADH‐TR shows preserved fiber typing, and there are several dark‐staining fibers (VI) The cytochrome oxidase stain (COX) also highlights the population of dark staining fibers, but there are no COX‐negative fibers (VII). Scale bar: A–D = 50 μm. (E) Muscle MRI of the lower limbs from Family 3 (P10) aged 8 months: Showing generalized atrophy in the absence of muscle fat infiltration. [Colour figure can be viewed at wileyonlinelibrary.com]

The affected individuals are six males and seven females, all of whom were born at full term, with all but two (P10 and P11) conceived from consanguineous unions (Fig. 1A). Eleven affected individuals are alive with a mean current age of 10 ± 6 years (range 4–24), and two individuals died at the ages of 11 months and 30 years old due to respiratory insufficiency and choking, respectively. Antenatal history was unremarkable in all but 3/13 persons who displayed decreased foetal movements.

Tables 1 and 2, Table S1, and Videos [Link], [Link] provide details on the clinical phenotype of the present cohort. The disease onset ranged from prenatal (3/13) to pre‐schooler age (mean age 16 ± 9.4 months, range 4–36). Delayed motor milestones (10/13), weak cry (3/13) and muscle weakness (3/13) were the first presenting symptoms. Mean ages were 9.7 ± 1.7 months for sitting, 1.7 ± 0.3 years for walking, and 9.9 ± 2.9 months for first words. Proximal or generalized muscle weakness (12/13) with the mean onset age of 2.7 ± 2.7 years (range 0.6–11), respiratory insufficiency (8/13) requiring non‐invasive ventilation (6/13) and tracheostomy (2/13), joint contractures (7/12), congenital or infantile‐onset bilateral severe sensorineural hearing loss (6/13) (only present in Families 1, 3, 4, and 5, while in the rest of the persons the absence of sensorineural hearing loss was clinically and objectively confirmed), and gastrointestinal issues (4/13) were among the commonly reported symptoms throughout the disease course (Tables 1 and 2). The age of onset for respiratory insufficiency in the affected individuals ranged between 7 months to 20 years old. Only one female person has reached puberty, and at 30 years old, she does not have clinical signs of primary ovarian insufficiency, while follicle‐stimulating hormone levels were reported to be high in P11, aged 12 years old. Six affected individuals lost ambulation at the mean age of 8.1 ± 4.7 years. Ninety‐two percent (12/13) of individuals displayed slow rates of disease progression. One person (P10) demonstrated improvement in motor abilities and dysphagia symptoms in childhood. Older individuals in our series tended to have a more severe phenotype reflecting the progressive nature of the disease in most instances.

**Table 2:** Frequency of the clinical features in the *GGPS1* cohorts.

Features	Current report	All reported cases
Decreased foetal movements	3/13	4/14
Birth OFC ≤25 percentile	6/10	6/10
Manifestation with delayed milestones	10/13	12/15
Slow progression	12/13	25/26
Failure to thrive	3/12	11/20
Hearing loss	6/13	18/26
Generalized or proximal muscle weakness	12/13	25/26
Respiratory insufficiency	8/13	16/23
Non‐invasive ventilation	6/13	10/17
Muscular atrophy	12/12	12/12
Myalgia	6/10	6/12
Muscle stiffness	6/12	6/12
Reduced or absent tendon reflexes	12/12	12/12
Calf hypertrophy	4/12	4/12
Joint contractures	7/12	11/23
Progressive scoliosis	7/11	15/21
Waddling gait	4/11	4/11
Broad‐based gait	2/11	2/11
Loss of ambulation	6/12	11/25
Elevated creatine kinase	10/10	19/19
Mortality	2/13	2/26

On the most recent examination, a mean age of the affected individuals was 11.5 ± 7.8 years (range 4.6–29). They had normal cognition (0/13) with short stature (8/13) and progressive scoliosis (7/11). Neuromuscular examination revealed hypotonia (10/13), proximal or generalized muscle weakness (12/13), and muscular atrophy (12/12) accompanied with stiffness (6/12), calf hypertrophy (4/12), hyporeflexia/areflexia (12/12), and myalgia (6/10). Gait was waddling in 4/11 and broad‐based in 2/11 persons (Tables 1 and 2). Electromyography findings were compatible with myopathy in 8/13 and elevated creatinine kinase (CK) (1594‐27000 U/L) was registered in 10/10 individuals. Lower limb muscle magnetic resonance imaging in Family 3 (P10), performed at the age of 8 months old, showed generalized muscle atrophy without fatty infiltration (Fig. 1E). Muscle biopsy from the quadriceps at age 8 months old in P10 showed type 1 fiber predominance and central nucleation with Z line streaming and mini‐cores evident on electron microscopy (Fig. 1D, I–IV). Muscle biopsy from the biceps in Family 4 (P11) showed fatty infiltration with mitochondrial changes (Fig. 1D, V–VII), whereas in Family 5 it showed a dystrophic pattern.

On exome sequencing, an ultra‐rare homozygous missense variant in exon 4 of *GGPS1* c.269A > G, p.(Asn90Ser) (NM_004837.4) residing within a 9.77 Mb region of homozygosity was identified in Family 1 (Fig. 1A–C, Figure S1). The p.(Asn90Ser) variant is located in a highly conserved GGPS1 protein region. The variant is absent in gnomAD and a number of large publicly available databases apart from 2 heterozygous alleles from the Centogene and TOPMed databases, and is predicted to be damaging by multiple in silico tools. (Table S2). The variant segregated with the phenotype within the family (Fig. 1A). Both Family 1 and Family 5 from Tucker et al. (2020) carried the same recurrent homozygous *GGPS1* c.269A > G; p.(Asn90Ser) variant. An ultra‐rare homozygous missense variant in exon 4 of *GGPS1* c.439A > G, p.(Met147Val) (NM_004837.4) residing within a 9 Mb region of homozygosity was identified in Family 2 (Fig. S1). This variant is located in a highly conserved GGPS1 protein region with predicted‐damaging scores on various in silico tools (Table S2). The variant segregated with the phenotype within the family (Fig. 1A).

Based on the extremely rare frequency of the identified *GGPS1* variants (PS4, PM2), co‐segregation in two unrelated families with multiple affected individuals for the *GGPS1* c.269A > G, p.(Asn90Ser) variant (PP1 moderate) and segregation in multiple affected family members from different branches for the *GGPS1* c.439A > G, p.(Met147Val) variant (PP1 moderate), as well as predicted‐deleterious effect from multiple in silico tools, we classified both variants from Family 1 and 2 as likely pathogenic according to the American College of Medical Genetics and Genomics (ACMG) guidelines for the interpretation of sequence variants.^9^

Two ultra‐rare missense variants in exon 4 of *GGPS1* were identified, in *trans*, in the proband of Family 3 (P10), c.196A > C p.(Ile66Leu) and c.545T > C p.(Leu182Pro) (Fig. 1). Both variants are absent from publicly available databases and are conserved across species (Table S2). The maternally inherited variant, c.196A > C p.(Ile66Leu), affects the alpha 3 helix of GGPPS whilst the paternally inherited variant affects the alpha 7 helix of the protein. Both implicated residues lie within the enzymes central catalytic barrel. The c.196. A > C p.(Ile66Leu) variant lies between two magnesium ion cofactor ligand bindings sites and the c.545T > C p.(Leu182Pro) variant lies near to the dimethylallyl diphosphate substrate binding sites within the GGPPS protein (Fig. S2). The individual's phenotype was felt to be highly specific for *GGPS1* related muscular dystrophy (Table 1). Owing to the rarity of the variants, and lack of further segregation and functional study data, these variants are classified as of uncertain significance according to ACMG guidelines. However, given the affected person's phenotype and proximity of the affected residues to key domains within GGPPS we feel they can be considered disease causing in P10.

An ultra‐rare missense variant c.770T > G; p.(Phe257Cys) was identified in Family 4. The variant is absent from all publicly available databases, is conserved across species, and is predicted damaging from a variety of in silico tools (Table S2). The variant segregated with the phenotype within the family (Fig. 1A).

Expression patterns of *Ggps1* in the mouse inner ear, skeletal muscle, and female gonad at different embryonic and postnatal stages were studied using single‐cell RNA sequencing. Single‐cell RNA sequencing of the cochlear epithelium in the E14, P1, and P7 mouse shows *Ggps1* with a sustained, diffuse expression at these timepoints (Fig. S3). Global view of *Ggps1* expression in the E14.5 female gonad showed clusters most densely in the germ cells and embryonic gonad cells (Fig. S4), whereas neonatal skeletal muscle showed several clusters reflecting comparatively lower expression, for example, in clusters representing stromal cells and chondrocytes (Fig. S5).

## Discussion

Protein prenylation is an important downstream part of the mevalonate pathway, which is a pivotal process for the synthesis of specialized lipids that play a crucial role in cellular processes at all levels.^1^, ^2^, ^10^ The mevalonate pathway has recently been suggested to be a novel pathway for muscular dystrophy, hearing loss, and infertility.^2^ Evidence for this comes from the description of 11 individuals from 6 independent families presenting with early onset muscular dystrophy combined with congenital sensorineural hearing loss and primary ovarian insufficiency in females. All but one affected individual in the report by Foley et al. (2020)^2^ had congenital sensorineural hearing loss, and all 3 postpubertal females had a laboratory‐confirmed primary ovarian failure. Andrological examinations on the postpubertal male individuals in the Foley et al. (2020)^2^ report were not available, but none of the 5 male adult individuals has had children. Although a combination of sensorineural hearing loss and muscular dystrophy or myopathy has been described in early‐onset facioscapulohumeral dystrophy and Vici syndrome, and an association of early‐onset sensorineural hearing loss and primary ovarian insufficiency without muscular dystrophy have been recognized in Perrault syndrome, a constellation of all these three features had not been previously recognized prior to the Foley et al. (2020) study.^2^ Disease course, in particular proximal muscle weakness, was progressive in the series by Foley et al. (2020)^2^ with some interfamilial variability in the rates of deterioration. When comparing the description and course of muscle clinical signs in these previously described affected individuals with expression in the neonatal mouse muscle, there are hints of relatively low *Ggps1* expression at this stage compared to the female gonad from E14.5 and we cannot exclude that expression in the muscle increases throughout later development. Muscle histopathology findings in the cohort described by Foley et al. (2020)^2^ were dystrophic with variable evidence of regenerating fibers, internal nucleation, and core‐like regions.

Biallelic pathogenic missense *GGPS1* variants identified in Foley et al. (2020)^2^ have been shown to moderately impair the enzymatic activity of geranylgeranyl diphosphate synthase (GGPPS) (only for 50%) in vitro, and the possible effects of these missense variants on GGPPS function have been speculated to be subtle, putatively including the impairment of the dynamic subcellular localization of the enzyme for cell‐type‐specific processes, particularly in muscle, ovary, and the inner ear. It had been suggested that the localization of the missense *GGPS1* variants residing outside of the catalytic core of the enzyme could account for the very specific and recognizable clinical phenotype. However, the variants reported in P10 (Family 3) co‐locate within the catalytic core of GGPPS and implicate impaired substrate and co‐factor binding as potential pathogenic mechanisms, resulting in a phenotype consistent with other reported individuals.

Family 2 in our cohort presents hitherto the largest family (6 affected) with biallelic *GGPS1* variants reported. Affected individuals from our report replicated most of the previously described clinical features associated with *GGPS1* defects. This includes muscular dystrophy with progressive proximal muscle weakness and episodes of variably elevated CK, scoliosis, and respiratory insufficiency. Muscle histopathology findings were also variable but largely consistent with those previously reported by Foley et al. (2020),^2^ including dystrophic changes, regenerating fibers, core‐like lesions, internal nucleation, and mitochondrial changes. The lack of dystrophic histopathology and fat infiltration observed in the muscle biopsy and lower limb MRI of Family 3 (P10) may reflect the early age (8 months) at which these investigations were performed, compared to other reported persons. The families described in our report display inter‐ and intra‐familial variability with respect to hearing loss, which was detected only in all affected siblings from Family 1, the probands from Families 3 and 4, and one affected sibling from Family 5. This could suggest that hearing loss might be a variable feature of *GGPS1‐*associated muscular dystrophy and is an interesting observation in light of its diffuse expression in the embryonic and postnatal mouse inner ear. Since most of the subjects with unimpaired hearing in the current report were from Family 2, carrying the *GGPS1* c.439A > G, p.(Met147Val) variant, we cannot exclude the possibility of a variant‐specific effect. Additionally, our data shows that primary ovarian insufficiency might also be a variable feature of *GGPS1* deficiency, despite strong *Ggps1* expression in relevant cells in the embryonic mouse female gonads. Although it is difficult to determine the exact molecular cause of the observed clinical variability, we can hypothesize a possible link with localisation of the *GGPS1* variants within the gene.

One possible limitation of the present study is the absence of in vitro enzyme activity measurements. However, taking into account the findings from the study by Foley et al. (2020),^2^ one could expect that the impact of the missense variants from our study on GGPPS function could be subtle as well, with an insignificant reduction of the enzymatic activity of GGPPS. All missense *GGPS1* variants c.269A > G, p.(Asn90Ser), c.439A > G, p.(Met147Val), c.196A > C p.(Ile66Leu), c.545 T > C p.(Leu182Pro) and c.770 T > G p(Phe257Cys) reported in the current study are ultra‐rare across several large genetic databases including 1,314,000 alleles (Table S2) and uniformly predicted to be damaging in various in silico prediction tools supporting their disease‐causing roles.

Collectively, this report consolidates the disease‐causing role of biallelic variants in *GGPS1*, demonstrates that hearing loss and ovarian insufficiency might be variable features of the *GGPS1*‐associated muscular dystrophy and implicates impaired substrate and co‐factor binding as potential pathogenic mechanisms in this novel form of congenital muscular dystrophy.

## Conflict of Interest

None of the authors has any conflict of interest to disclose.

## Supporting information


**Figure S1** Region of homozygosity around the variant of interest indicated by a red box.^11^

**Figure S2** 3D protein modeling of the *GGPS1* variants in Family 3 (P10).

**Figure S3** Expression of *Ggps1* in the mouse cochlea through single‐cell RNA‐sequencing data.

**Figure S4** Expression of *Ggps1* in the mouse embryonic (E14.5) gonad through single‐cell RNA‐sequencing data.

**Figure S5** Expression of *Ggps1* in mouse neonatal leg muscle through single‐cell RNA‐sequencing data.

**Table S1** Extended clinical features of the cases with biallelic *GGPS1* variants.

**Table S2** Population frequencies and in silico pathogenicity predictions for *GGPS1* variants reported in this study.

**Video S1** Shows P1 with a waddling gait, lower limb weakness, and inverted feet.

**Video S2** Shows P4 who is unable to stand unassisted and walk due to the lower limb weakness.

**Video S3** Shows P5 with a suggestion of Gower's sign and waddling gait.

**Video S4** shows P6 with proximal lower limb weakness.

**Video S5** shows P7 with positive Gower's sign.

**Video S6** shows P8 with positive Gower's sign.

## Data Availability

The datasets generated during and/or analyzed during the current study are available from the corresponding author on reasonable request.

## References

[1] Wang M , Casey PJ . Protein prenylation: unique fats make their mark on biology. Nat Rev Mol Cell Biol. 2016;17(2):110‐122. 26790532 10.1038/nrm.2015.11

[2] Foley AR , Zou Y , Dunford JE , et al. GGPS1 mutations cause muscular dystrophy/hearing loss/ovarian insufficiency syndrome. Ann Neurol. 2020;88(2):332‐347. 32403198 10.1002/ana.25772 PMC7496979

[3] Tucker EJ , Rius R , Jaillard S , et al. Genomic sequencing highlights the diverse molecular causes of Perrault syndrome: a peroxisomal disorder (PEX6), metabolic disorders (CLPP, GGPS1), and mtDNA maintenance/translation disorders (LARS2, TFAM). Hum Genet. 2020;139(10):1325‐1343. 32399598 10.1007/s00439-020-02176-w

[4] Makrythanasis P , Maroofian R , Stray‐Pedersen A , et al. Biallelic variants in KIF14 cause intellectual disability with microcephaly. Eur J Hum Genet. 2018;26(3):330‐339. 29343805 10.1038/s41431-017-0088-9 PMC5839044

[5] Pais LS , Snow H , Weisburd B , et al. A web‐based analysis and collaboration tool for rare disease genomics. Hum Mutat. 2022;43(6):698‐707. 35266241 10.1002/humu.24366 PMC9903206

[6] Natera‐de Benito D , Jurgens JA , Yeung A , et al. Recessive variants in COL25A1 gene as novel cause of arthrogryposis multiplex congenita with ocular congenital cranial dysinnervation disorder. Hum Mutat. 2022;43(4):487‐498. 35077597 10.1002/humu.24333 PMC8960342

[7] Orvis J , Gottfried B , Kancherla J , et al. gEAR: gene expression analysis resource portal for community‐driven, multi‐omic data exploration. Nat Methods. 2021;18(8):843‐844. 34172972 10.1038/s41592-021-01200-9 PMC8996439

[8] Han X , Wang R , Zhou Y , et al. Mapping the mouse cell atlas by microwell‐seq. Cell. 2018;172(5):1091‐107.e17. 29474909 10.1016/j.cell.2018.02.001

[9] Richards S , Aziz N , Bale S , et al. Standards and guidelines for the interpretation of sequence variants: a joint consensus recommendation of the American College of Medical Genetics and Genomics and the Association for Molecular Pathology. 10.1038/gim.2015.30 PMC4544753 25741868

[10] Jeong A , Suazo KF , Wood WG , Distefano MD , Li L . Isoprenoids and protein prenylation: implications in the pathogenesis and therapeutic intervention of Alzheimer's disease. Crit Rev Biochem Mol Biol. 2018;53(3):279‐310. 29718780 10.1080/10409238.2018.1458070 PMC6101676

[11] Quinodoz M , Peter VG , Bedoni N , et al. AutoMap is a high performance homozygosity mapping tool using next‐generation sequencing data. Nat Commun. 2021;12(1):518. 33483490 10.1038/s41467-020-20584-4 PMC7822856

[12] Kavanagh KL , Dunford JE , Bunkoczi G , Russell RGG , Oppermann U . The crystal structure of human geranylgeranyl pyrophosphate synthase reveals a novel hexameric arrangement and inhibitory product binding. J Biol Chem. 2006;281(31):22004‐22012. 16698791 10.1074/jbc.M602603200

[13] Chang TH , Guo RT , Ko TP , Wang AH , Liang PH . Crystal structure of type‐III geranylgeranyl pyrophosphate synthase from Saccharomyces cerevisiae and the mechanism of product chain length determination. J Biol Chem. 2006;281(21):14991‐15000. 16554305 10.1074/jbc.M512886200

