# Supplementary material for: GGPS1 ‐associated muscular dystrophy with and without hearing loss
Source: Ann Clin Transl Neurol. 2022 Jul 23;9(9):1465–74. doi: 10.1002/acn3.51633 (PMC9463955; doi:10.1002/acn3.51633)

Homozygous Regions for p.(Asn90Ser)
Total = 147.49 Mb (autosomes)

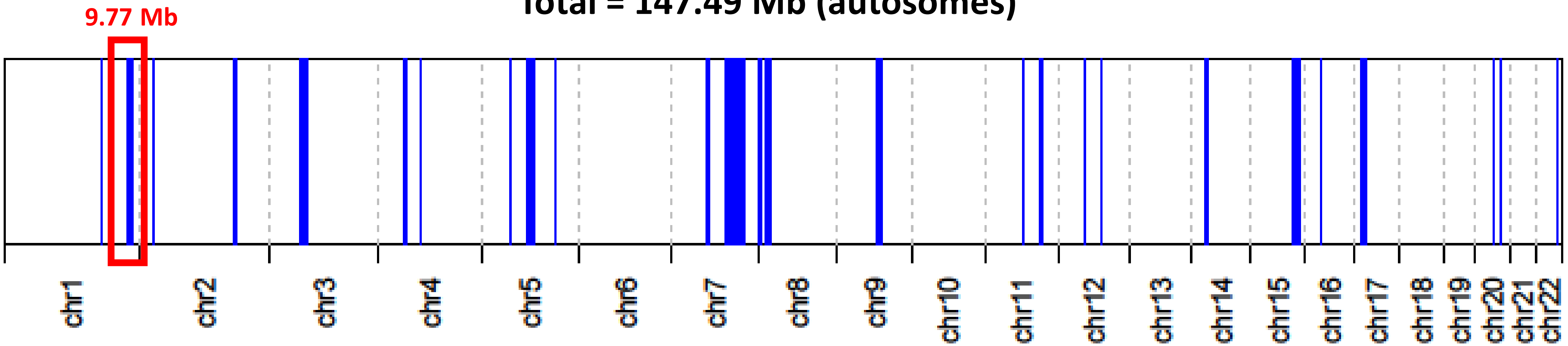

Homozygous Regions for p.(Met147Val)
Total = 461.19 Mb (autosomes)

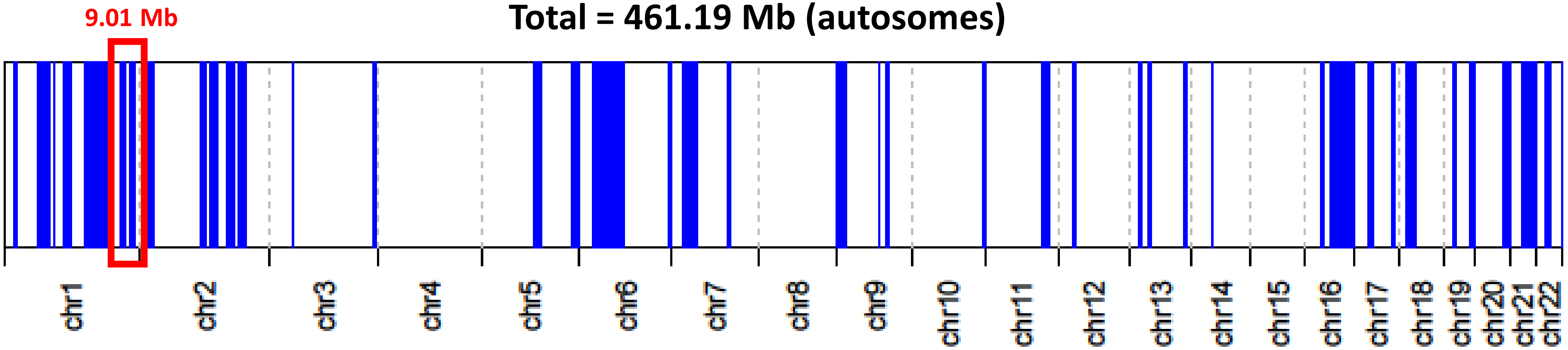

Supplement: Supplementary file 1 — Figure S1 Region of homozygosity around the variant of interest indicated by a red box. 11 [file ACN3-9-1465-s009.pdf]
