# Supplementary figures and images for: GGPS1 ‐associated muscular dystrophy with and without hearing loss
Source: Ann Clin Transl Neurol. 2022 Jul 23;9(9):1465–74. doi: 10.1002/acn3.51633 (PMC9463955; doi:10.1002/acn3.51633)

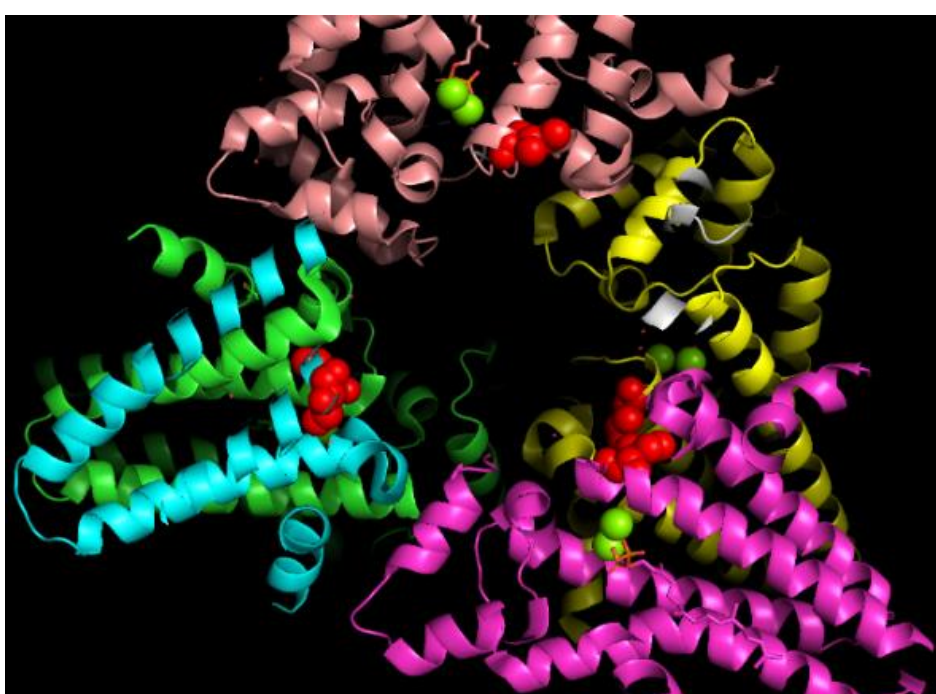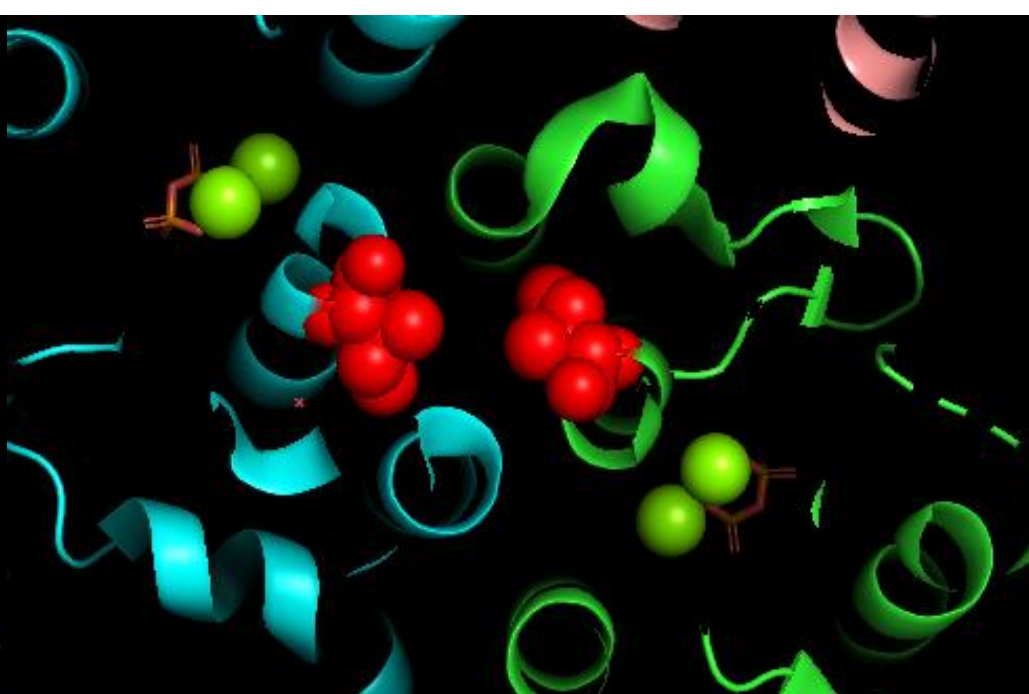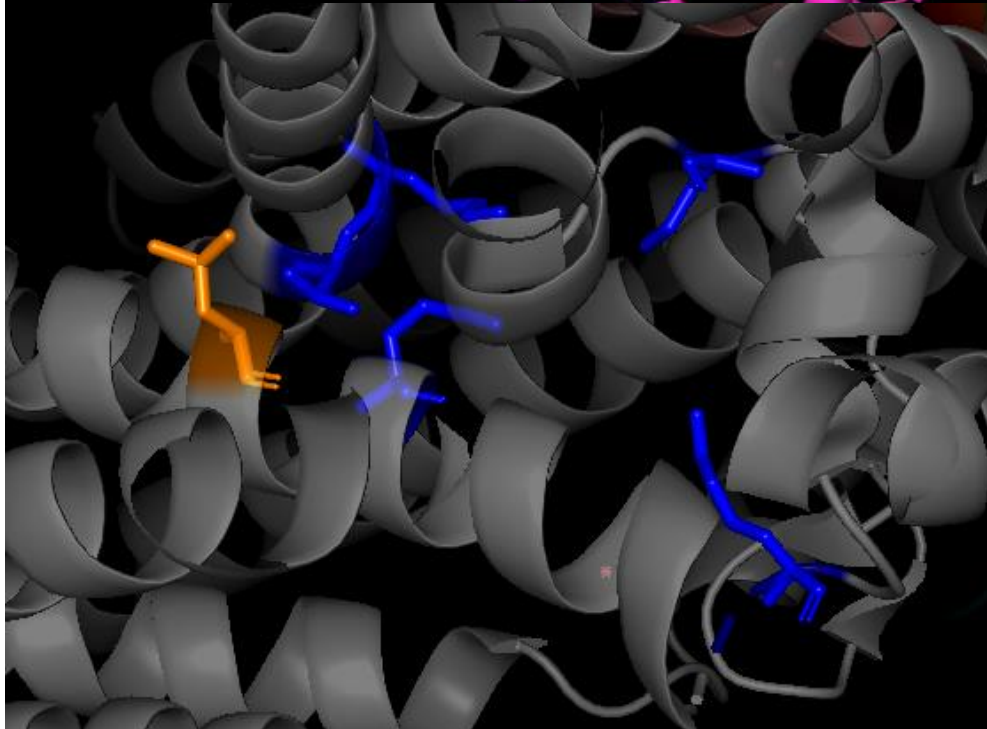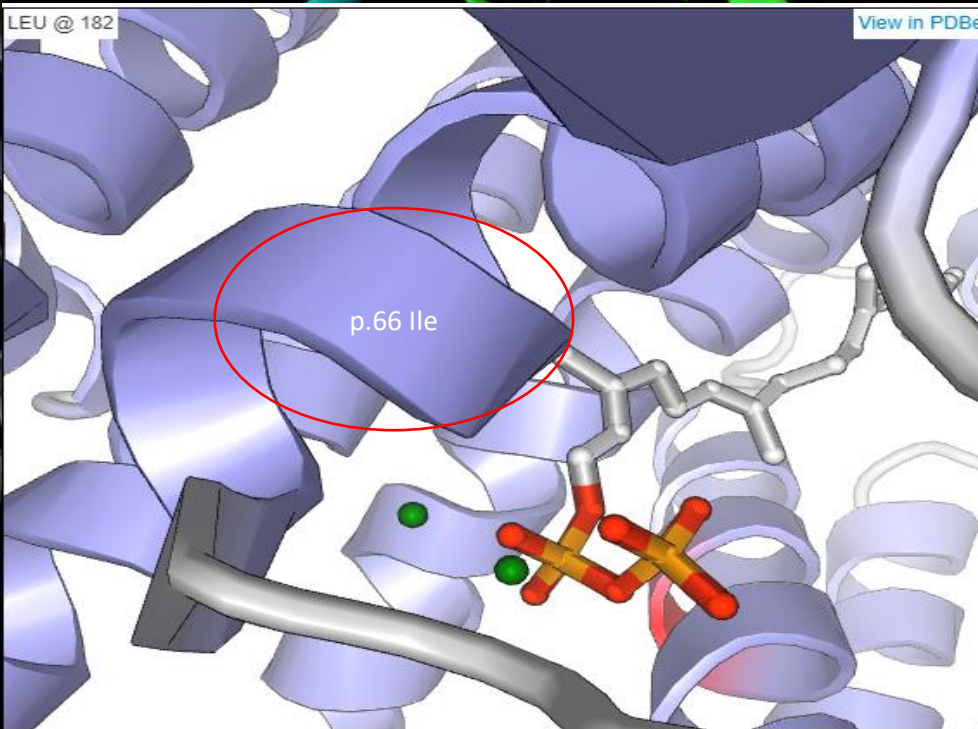

Supplement: Supplementary file 2 — Figure S2 3D protein modeling of the GGPS1 variants in Family 3 (P10). [file ACN3-9-1465-s007.pdf]

## E14.5 mouse female gonad

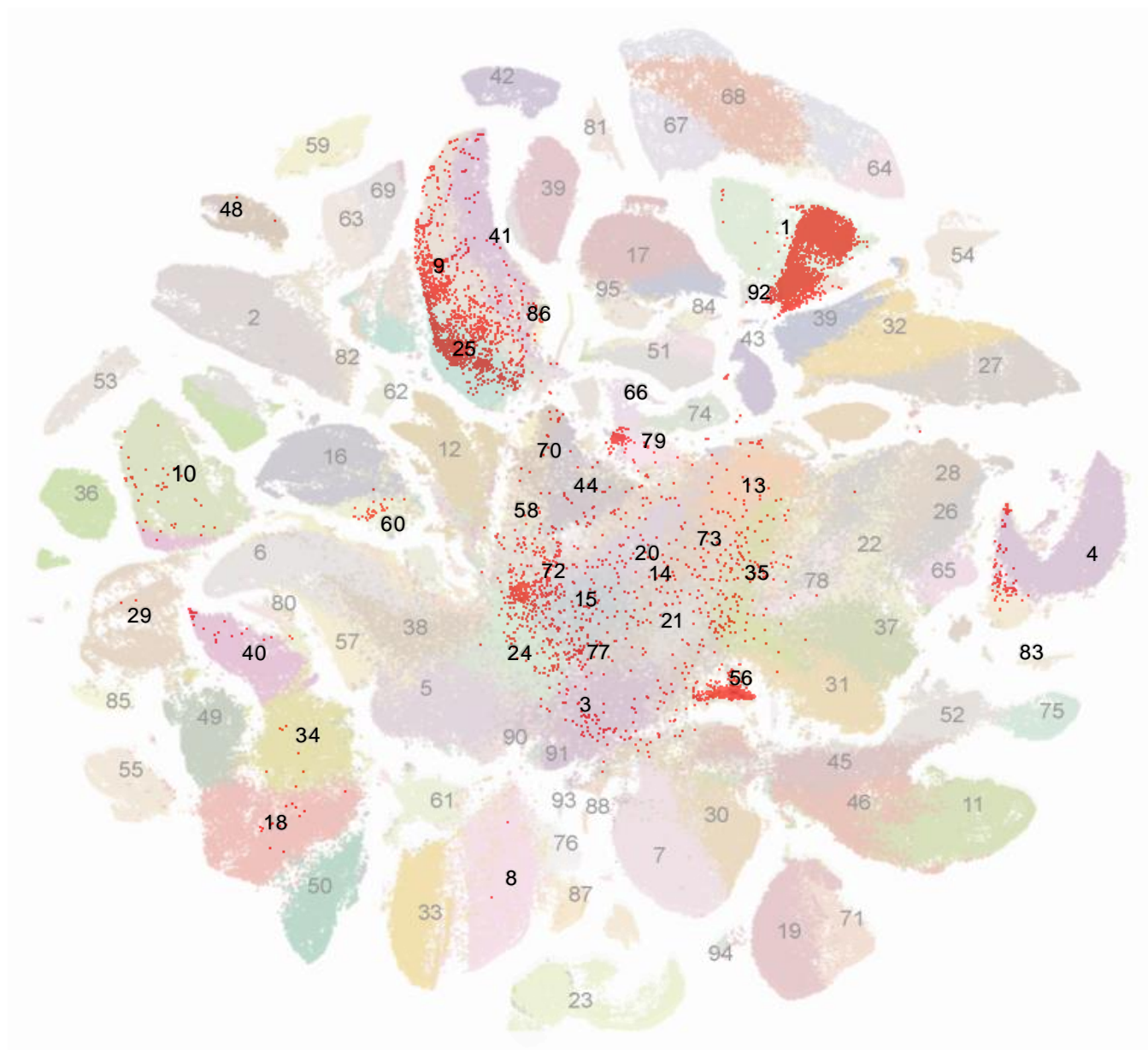

Supplement: Supplementary file 4 — Figure S4 Expression of Ggps1 in the mouse embryonic (E14.5) gonad through single‐cell RNA‐sequencing data. [file ACN3-9-1465-s011.pdf]

Neonatal mouse muscle

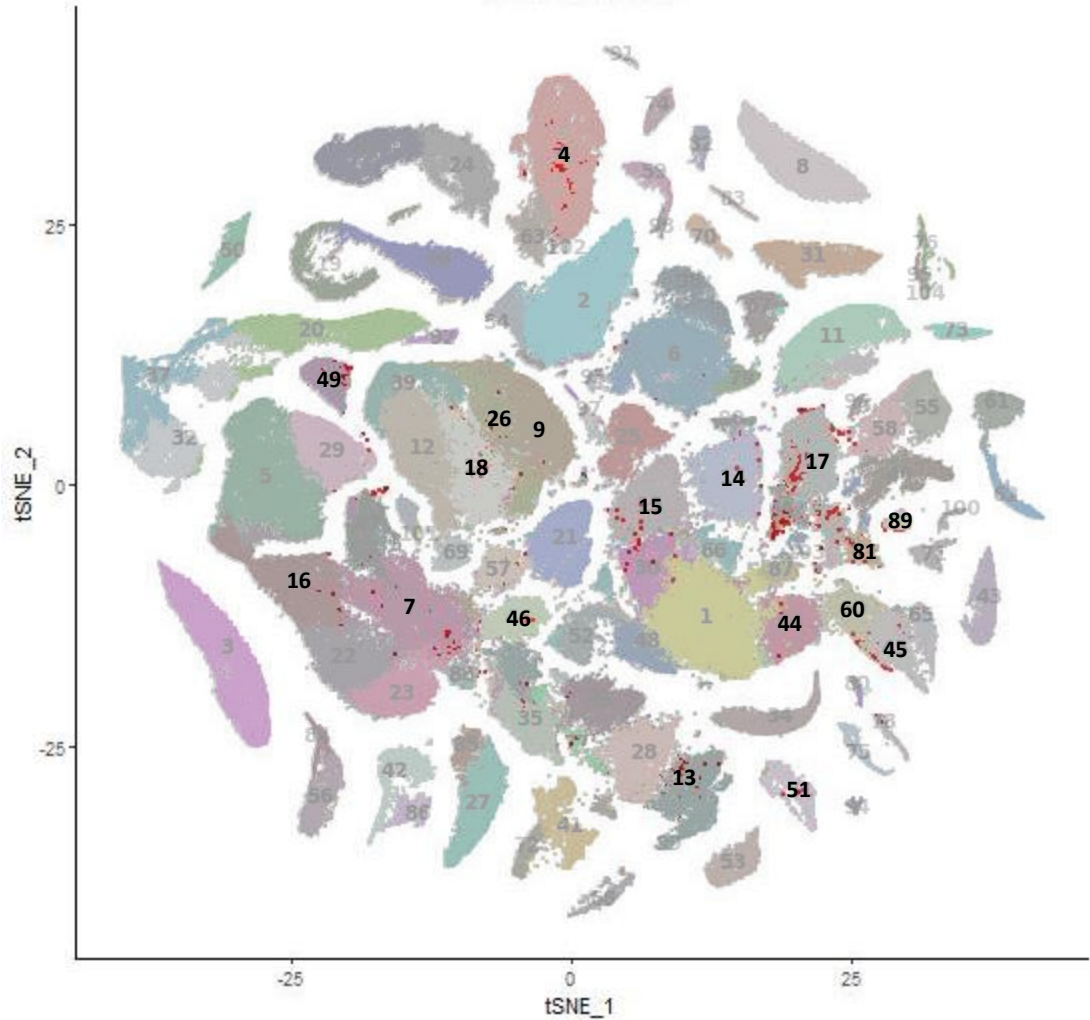

Supplement: Supplementary file 5 — Figure S5 Expression of Ggps1 in mouse neonatal leg muscle through single‐cell RNA‐sequencing data. [file ACN3-9-1465-s005.pdf]
