# Supplementary material for: GGPS1 ‐associated muscular dystrophy with and without hearing loss
Source: Ann Clin Transl Neurol. 2022 Jul 23;9(9):1465–74. doi: 10.1002/acn3.51633 (PMC9463955; doi:10.1002/acn3.51633)
Supplement: Supplementary file 3 — Figure S3 Expression of Ggps1 in the mouse cochlea through single‐cell RNA‐sequencing data. [file ACN3-9-1465-s003.pdf]

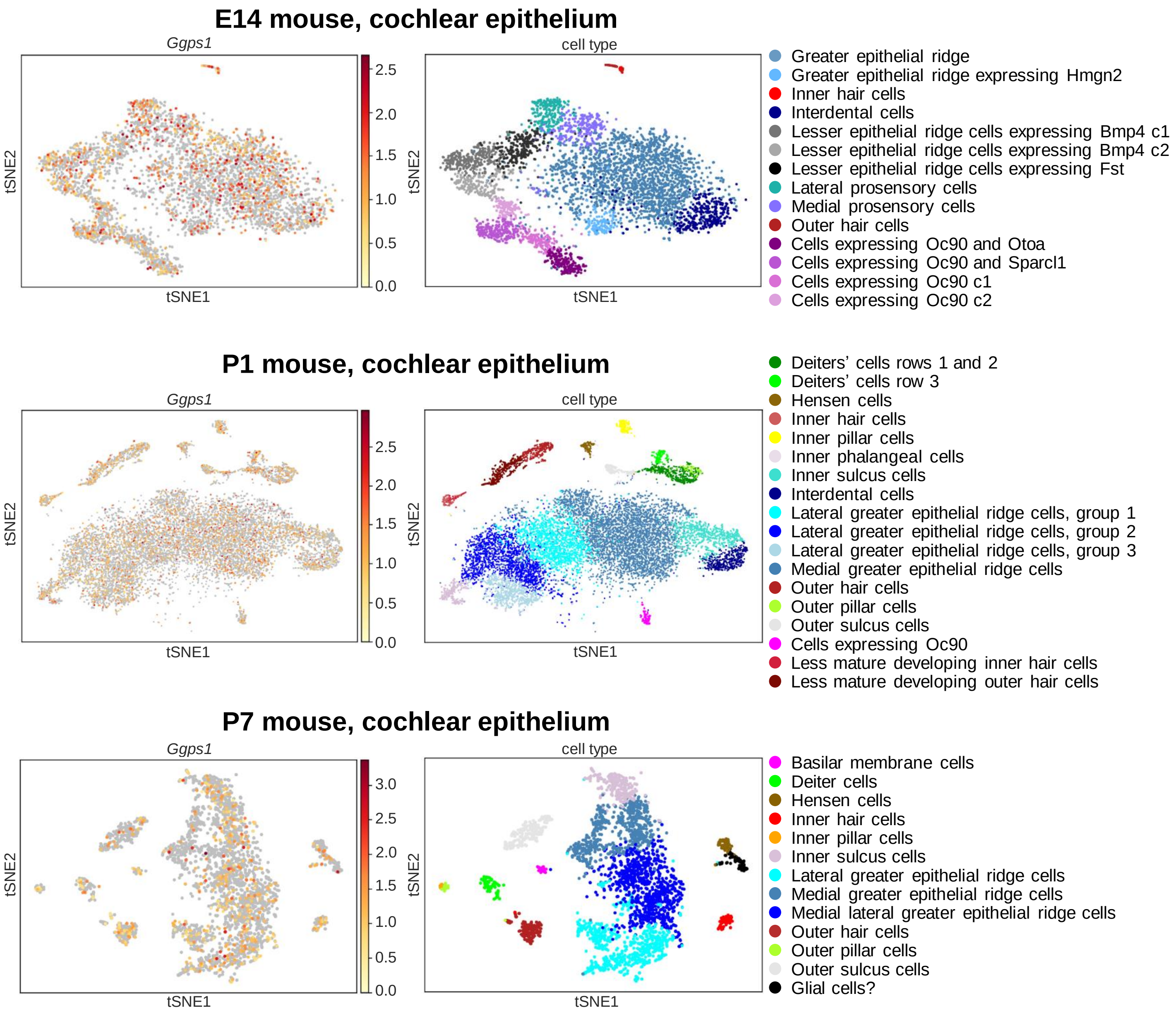

E14 mouse, cochlear epithelium
Ggps1
cell type
tSNE2
tSNE1
0.0
0.5
1.0
1.5
2.0
2.5
Greater epithelial ridge
Greater epithelial ridge expressing Hmgn2
Inner hair cells
Interdental cells
Lesser epithelial ridge cells expressing Bmp4 c1
Lesser epithelial ridge cells expressing Bmp4 c2
Lesser epithelial ridge cells expressing Fst
Lateral prosensory cells
Medial prosensory cells
Outer hair cells
Cells expressing Oc90 and Otoa
Cells expressing Oc90 and Sparcl1
Cells expressing Oc90 c1
Cells expressing Oc90 c2
P1 mouse, cochlear epithelium
Ggps1
cell type
tSNE2
tSNE1
0.0
0.5
1.0
1.5
2.0
2.5
Deiters' cells rows 1 and 2
Deiters' cells row 3
Hensen cells
Inner hair cells
Inner pillar cells
Inner phalangeal cells
Inner sulcus cells
Interdental cells
Lateral greater epithelial ridge cells, group 1
Lateral greater epithelial ridge cells, group 2
Lateral greater epithelial ridge cells, group 3
Medial greater epithelial ridge cells
Outer hair cells
Outer pillar cells
Outer sulcus cells
Cells expressing Oc90
Less mature developing inner hair cells
Less mature developing outer hair cells
P7 mouse, cochlear epithelium
Ggps1
cell type
tSNE2
tSNE1
0.0
0.5
1.0
1.5
2.0
2.5
3.0
Basilar membrane cells
Deiter cells
Hensen cells
Inner hair cells
Inner pillar cells
Inner sulcus cells
Lateral greater epithelial ridge cells
Medial greater epithelial ridge cells
Medial lateral greater epithelial ridge cells
Outer hair cells
Outer pillar cells
Outer sulcus cells
Glial cells?
