# Supplementary material for: GGPS1 ‐associated muscular dystrophy with and without hearing loss
Source: Ann Clin Transl Neurol. 2022 Jul 23;9(9):1465–74. doi: 10.1002/acn3.51633 (PMC9463955; doi:10.1002/acn3.51633)
Supplement: Supplementary file 6 — Table S1 Extended clinical features of the cases with biallelic GGPS1 variants. [file ACN3-9-1465-s004.docx]

| **Supplementary Table 1. Extended clinical features of the affected individuals with biallelic GGPS1 variants** | | | | | | | | | | | | | | | | |  |
| --- | --- | --- | --- | --- | --- | --- | --- | --- | --- | --- | --- | --- | --- | --- | --- | --- | --- |
|  |  | **Family 1** | | | **Family 2** | | | | | | **Family 3** | **Family 4** | **Family 5 (from Tucker et al., 2020)** | | **Foley et al. 2020 (11 personss/6 families)** | **Tucker et al.2020 (4 persons/2 families)** |  |
|  | **Person** | P1 | P2 | P3 | P4 | P5 | P6 | P7 | P8 | P9 | P10 | P11 | P 12 | P 13 |  |  |  |
| **Variant details** | Variant type | Homozygous | | | Homozygous | | | | | | Compound heterozygous | Homozygous | Homozygous | | Homozygous/compound heterozygous | Homozygous |  |
|  | Variant at the cDNA level (NM_004837.4) | c.269A>G | | | c.439A>G | | | | | | c.196A>C and c.545T>C | c.770T>G | c.269A>G | c.269A>G | (c.860A > G;865C > G), (c.127C > T; 865C > G), c.866G > A, c.854 T > G | c.782G>A, c.269A>G |  |
|  |  |  |  |  |  |  |  |  |  |  |  |  |  |  |  |  |  |
|  |  |  |  |  |  |  |  |  |  |  |  |  |  |  |  |  |  |
|  | Variant at the protein level | p.(Asn90Ser) | | | p.(Met147Val) | | | | | | p.(Ill66Leu) and p.(Leu182Pro) | p.(Phe257Cys) | p.(Asn90Ser) | p.(Asn90Ser) | [p.(Tyr259Cys); p.(Arg261Gly)], [p.(Pro15Ser); p. (Arg261Gly)], p.(Arg261His), p.(Phe257Cys) | p.(Arg261His), p.(Asn90Ser) |  |
|  |  |  |  |  |  |  |  |  |  |  |  |  |  |  |  |  |  |
|  |  |  |  |  |  |  |  |  |  |  |  |  |  |  |  |  |  |
| **Epidemiology** | Sex | F | F | F | M | M | M | M | M | F | F | F | M | F | F-6; M - 5 | F -3, M -1 |  |
|  | Consanguinity | + | + | + | + | + | + | + | + | + | - | + | + | + | 1 family-no, 5 families -NA | NA |  |
|  | Family history | + | + | + | + | + | + | + | + | + | - | - | - | - | 3 | + |  |
|  | Current age | 11y.o. | 11 m.o. (Died) | 8y.o. | 23.5 y.o. | 4y 8m.o. | 5y 7m.o. | 4y .o. | 5y.o. | 30 y.o. (Died) | 8 y.o | 12 y.o. | 20 y.o. | 8.5 y.o. | 31, 29,22,46,45,44,14,21,22,11,8 (y.o.) | 36,39,7 (y.o.) |  |
|  |  |  |  |  |  |  |  |  |  |  |  |  |  |  |  |  |  |
|  | Age of death | Alive | 11 m.o. | Alive | Alive | Alive | Alive | Alive | Alive | 30 y.o. | Alive | Alive | Alive | Alive | NA | Alive |  |
| **Birth** | Decreased foetal movements | + | + | + | - | - | - | - | - | - | - | - | - | - | +(1) | NA |  |
|  | Weeks of gestation | Term | Term | Term | 40 weeks | 39 weeks | 40 weeks | 38 weeks | 39 weeks | 40 weeks | Term | NA | NA | Term | 1 - 29 weeks, | NA |  |
|  | Head circumference at birth | 35 cm, 75^th^ percentile | 36 cm, 75^th^ percentile | 33 cm, 25^th^ percentile | 35cm, 50^th^ percentile | 34 cm, 25^th^ percentile | 34.5cm, 25^th^ percentile | 33.5cm, 10^th^ percentile | 35cm, 50^th^ percentile | 33cm, 25^th^ percentile | NA | NA | NA | 33, 25^th^ percentile | NA | NA |  |
|  | Birth weight | 2.8 kg, 25^th^ percentile | 3.1 kg, 50^th^ percentile | 3.3 kg, 50^th^ percentile | 3.5 kg 50^th^, percentile | 3.2kg 50^th^ percentile | 3.3 Kg 50^th^ percentile | 2.9kg, 25^th^ percentile | 3.2kg, 50^th^ percentile | 3kg, 50^th^ percentile | 2.6kg (2-9^th^ centile) | NA | NA | 2.7kg, 25^th^ percentile | NA | NA |  |
|  |  |  |  |  |  |  |  |  |  |  |  |  |  |  |  |  |  |
|  | Birth length | 52 cm, 75^th^ percentile | 52 cm, 75^th^ percentile | 51 cm, 50^th^ percentile | 50cm, 50^th^ percentile | 49.5 cm, 50^th^ percentile | 50 cm, 50^th^ percentile | 48cm, 25^th^ percentile | 49cm, 25^th^ percentile | 48cm, 25^th^ percentile | NA | NA | NA | 47cm, 25^th^ percentile | NA | NA |  |
|  |  |  |  |  |  |  |  |  |  |  |  |  |  |  |  |  |  |
|  | Neonatal with a weak cry and a poor suck | + | + | + | - | - | - | - | - | - | - | - | - | - | + (1) | NA |  |
| **Medical history** | Age at onset | Prenatal | Prenatal | Prenatal | 1y 8m.o. | 8 m.o. | 6 m.o. | 1y 6m.o. | 2y.o. | 3y.o. | 7 months | 18 m.o. | Motor delay noted at 18 months | 4 months | Prenatal | Childhood |  |
|  | First presentation | Weak cry | Weak cry | Weak cry | Delayed walking | Delayed motor millstones | Muscle weakness /flaccidity /Delayed walking | inability to stand well from sitting position | Delayed walking | Muscle weakness | Hypotonia, muscle weakness. Respiratory insufficiency | Motor delay | Motor delay noted at 18 months | Motor delay | Hearing loss in cases with milder motor phenotypes, manifestations with motor delay in 2 persons | Hearing loss |  |
|  | Type of progression (rapid, moderate, slow) | Slow | Slow | Slow | Slow | Slow | Slow | Slow | Slow | Slow | Slow (progressive scoliosis with improvements in motor and swallowing function). | Slow | Moderate | Slow | Slow (11) | Slow (4) |  |
|  | Failure to thrive | + | NA | + | - | - | - | - | - | - | - | - | - | + | + (8) | NA |  |
|  | Sensorineural hearing loss | + | + | + | - | - | - | - | - | - | + Normal new-born hearing screen. Hearing loss detected at 4 years | + | - | + from birth | + (10), -(1) | + (3),- (1) |  |
|  | Progressive muscle weakness, age of onset | 1.5y.o. | NA | 1.5 y.o | 4y.o. | 2y.o. | 1y and 3m.o. | 1y and 6m.o. | 2y.o. | 3y.o. | 7 months | 19 m.o. | 11 y.o. | - | + (4), less severe weakness (7) | Mild (3 ) , severe (1) |  |
|  | Joint contractures | + | NA | + | + | - | - | - | - | + | - | + | + | + | + (4) | NA |  |
|  |  |  |  |  |  |  |  |  |  |  |  |  |  |  |  |  |  |
|  | Respiratory insufficiency, age of onset | +,13m.o. | +, 8 m.o. | +, 15 m.o. | + | - | - | - | - | +, 20 y.o. | +, 7m.o. | +, 10y.o. | +, 13y.o. | - | + (8) | + (1) |  |
|  |  |  |  |  |  |  |  |  |  |  |  |  |  |  |  |  |  |
|  | Non-invasive ventilation | + | + | + | - | - | - | - | - | + | + | - | + | - | +4 | + (1) |  |
|  | Tracheostomy | - | - | - | - | - | - | - | - | + | - | - | + | - | + (1) | + (1) |  |
|  | Intermittent episodes of diarrhoea accompanied by poor feeding | - | - | - | + | - | - | + | - | - | - | - | - | - | NA | NA |  |
|  | Gastrostomy tube | - | - | - | - | - | - | - | - | + | - | - | + | - | + (1) | NA |  |
|  | Primary ovarian insufficiency | NA | NA | NA | na | na | na | na | na | - | NA | Yes | na | - | + (3), uncertain to age (3) | + (2) |  |
|  | Cardiac involvement | - | NA | - | - | - | - | - | - | - | - | - | - | - | - |  |  |
|  | Main medical problem | Weakness | Respiratory insufficiency | Weakness | Severe muscle weakness, inability to walk, confined to wheel chair | Muscle Weakness | Muscle Weakness | Muscle weakness | Muscle weakness | Severe weakness, feeding difficulties and breathing defect | Four limb proximal and axial muscle weakness with respiratory insufficiency and progressive scoliosis. | Muscle weakness | Muscular deficit, feeding, breathing | Muscular deficit, low weight, SNHL | NA | Myopathy (1) |  |
|  | Loss of ambulation (age) | 7 y.o. | NA | 7 y.o. | 18y.o. | - | - | - | - | 17 y.o | NA | 9 y.o. | 11y.o. | - | + (5) 11yo, 13yo, 15,12,11 | NA |  |
| **Developmental stages** | Age of sitting | 1 y.o. | NA |  | 1y.o. | 1y.o. | 8m.o. | 8m.o. | 8m.o. | 9m.o. | 11 months | 8m.o. | NA | 9 months |  | NA |  |
|  | Age of walking | 2.5y.o. | NA | 2.5 y.o. | 2y 3m.o. | 1y and 9m.o. | 2y.o. | 1y 5m | 2y 2m | 2y.o. | 2 years | 18m.o. | 24 m.o. | 18 m.o. | 18m.o. | NA |  |
|  | Age of first words/Language abilities | 8 m.o. | 9 m.o. | 8 m.o. | 8m.o. | 9 m.o. | 9m.o. | 1y.o. | 9m.o. | 9m.o. | NA | 18m.o. | Normal | Few words. | NA | NA |  |
| **Physical examination** | Age at last examination | 11 y.o. | NA | 9 y.o. | 23.5y.o. | 4y and 8m | 5y and 7m.o. | 4 y.o. | 5y.o. | 29y.o. | 7 years | 12y.o. | 20 y.o. | 8 y.o. | 28, 26 | NA |  |
|  | Weight at last examination | NA | NA | NA | 65kg | 18kg | 19kg | 17kg | 16kg | 39.5kg | NA | NA | 42 at 20y | 14 |  | NA |  |
|  | Height at last examination | NA | NA | NA | 145cm | 102 cm | 110cm | 91cm | 92cm | 138cm | NA | NA | 157 at 16y | 113 cm |  | NA |  |
|  | Progressive scoliosis | - | NA | + | + | - | - | - | + | + | + | + | + | NA | + (8) | NA |  |
|  | Spinal fusion surgery | - | NA | - | - | - | - | - | - | - | - | - | + | - | + (1) | NA |  |
|  | Short stature | + | NA | + | + | - | - | + | + | + | 25th centile for age | NA | + | + | NA | NA |  |
| **Neurological examination** | Cranial nerves | Normal | Normal | Normal | Normal | Normal | Normal | Normal | Normal | Normal | Normal | Normal | Normal | Normal | NA | NA |  |
|  | Hypotonia | - | - | - | + | + | + | + | + | + | + | + | + | + | NA | NA |  |
|  | Muscle weakness | + | NA | + | + | + | + | + | + | + | + | + | + | + | + (1) | + (2) |  |
|  | Body distribution of muscular weakness | Leg/foot, pelvic girdle, trunk | Poor head control | Leg/foot, pelvic girdle, trunk | Pelvic girdle /shoulder girdle / back | Shoulder girdle/ Pelvic girdle | Shoulder girdle and pelvic girdle | Shoulder girdle and pelvic, girdle | Shoulder girdle and pelvic girdle | Generalized | Four limb proximal and axial muscle weakness | Axial and proximal | LL>UL | LL>UL | NA | NA |  |
|  | Pattern of muscular weakness | NA | NA | NA | Generalized | Proximal | Proximal | Proximal | Proximal | Generalized | LL>UL | Axial and proximal | LL > UL | LL>UL | NA | NA |  |
|  | Muscle hypertrophy | - | NA | - | - | Mild calf muscles | Calf muscles | Calf muscles | Calf muscles | - | - | - | - | - | NA | NA |  |
|  | Peripheral neuropathy | - | - | - | - | - | - | - | - | - | - | - | - | NA | NA | NA |  |
|  | DTRs | 0 | NA | 0 | 0 | ↓ | ↓ | ↓ | ↓ | 0 | ↓ | ↓ | ↓↓ | ↓↓ | NA | NA |  |
|  | Muscular atrophy | + | NA | + | + | + | + | + | + | + | + | + | + | + | NA | NA |  |
|  | Myalgia | - | NA | - | + | + | + | + | + | + | - | NA | NA | - | NA | + (2) |  |
|  | Stiffness | + | NA | + | + | - | - | - | - | + | - | NA | + | + | NA | NA |  |
|  | Gait | Broad-based | NA | Broad-based | Non ambulant | Waddling gait | Waddling gait | Normal gait | Waddling gait | Non ambulant | Unsteady | Non ambulant | NA | Waddling gait | Non-ambulant (5), NA (6) | NA |  |
| **Details on Hearing loss** | The age of onset | 11 m.o. | 6 m.o. | 6 m.o. | Intact | Intact | Intact | Intact | Intact | Intact | Normal new-born hearing screening. Abnormal hearing test at 4 years old | Not sure - childhood | Intact | From birth | From neonatal to childhood | Childhood |  |
|  | The type of hearing loss | SNHL | SNHL | SNHL | - | - | - | - | - | - | SNHL | SNHL | - | SNHL | SNHL | SNHL |  |
|  | The laterality and symmetry of the hearing loss | Bilateral | NA | Bilateral | - | - | - | - | - | - | Bilateral | Bilateral | - | Bilateral | Bilateral | Bilateral |  |
|  | The degree of hearing loss | Severe | NA | Severe | - | - | - | - | - | - | Severe | Severe | - | 80 dB on each side | NA | NA |  |
| **Investigations** | Elevated CK (age) | NA | NA | NA | 14y.o.: 2000 | 4y.o.: 6000 | 5y.o.: 6600 | 3y.o.: 27000 | 3y.o.: 12000 | 11y.o.: 5000 | +(7m.o 1594U/L, 4y.o 5490U/L) | 19m.o. | 7000 | 2000 | + (9) | NA |  |
|  | FSH | NA | NA | NA | na | na | na | na | na | NA | NA | High | NA | NA | 88.2IU/l, 50.3IU/l, 53.2IU/l | 60 IU/I, 35.8 IU/I |  |
|  | EMG | Normal | Normal | Normal | Myopathic picture | Myopathic picture | Myopathic | Myopathic | Myopathic | Myopathic | Myopathic | Myopathic | Normal | Normal | NA | NA |  |
|  | Muscle biopsy/ histochemistry | NA | NA | NA | Not done | No done | No done | No done | No done | No done | (8m.o) Type 1 fibre predominance, central nuclei, Z line streaming, mini-cores. | Fatty infiltration with mitochondrial changes | Dystrophic pattern | Dystrophic pattern | + (9) Dystrophic, with evidence of degeneration | NA |  |
|  |  |  |  |  |  |  |  |  |  |  |  |  |  |  |  |  |  |
|  | Muscle MRI | NA | NA | NA | Not done | Done | Done | Done | Done | Not done | (8m.o) generalised muscle atrophy without fatty infiltration. | NA | NA | NA | + (3) Fatty infiltration consistent with an underlying muscular dystrophy. | NA |  |

Abbreviations: P- Person; NA – not available; na- not applicable; y.o. – years old; m.o. – months old; LL- lower limbs; UL – upper limbs; SNHL – sensorineural hearing loss; FSG -follicle stimulating hormone; EMG- electromyography; MRI – magnetic resonance tomography; m – male; f- female; CK – creatine kinase; DTRs – deep tendon reflexes. dB – decibel.
