# Supplementary material for: GGPS1 ‐associated muscular dystrophy with and without hearing loss
Source: Ann Clin Transl Neurol. 2022 Jul 23;9(9):1465–74. doi: 10.1002/acn3.51633 (PMC9463955; doi:10.1002/acn3.51633)
Supplement: Supplementary file 7 — Table S2 Population frequencies and in silico pathogenicity predictions for GGPS1 variants reported in this study. [file ACN3-9-1465-s002.docx]

**Supplementary Table 2.** **Population frequencies and *in silico* pathogenicity predictions for *GGPS1* variants reported in this study**

| **Gene** | ***GGPS1*** | ***GGPS1*** | ***GGPS1*** | ***GGPS1*** | ***GGPS1*** |
| --- | --- | --- | --- | --- | --- |
| **Variant** | c.269A>G  p.(Asn90Ser) | c.439A>G  p.(Met147Val) | c.196A>C p.(Ill66Leu) | c.545T>C p.(Leu182Pro) | c.770T>G p(Phe257Cys) |
| **Transcript** | NM_004837.4 | NM_004837.4 | NM_004837.4 | NM_004837.4 | NM_004837.4 |
| **Rs-ID** | rs1012843795 | rs1237799550 | Absent | Absent | Absent |
| **gnomAD** | Absent | 1 heterozygous allele out of 251328 | Absent | Absent | Absent |
| **Iranome and GME** | Absent | Absent | Absent | Absent | Absent |
| **UK Biobank** | Absent | 2 heterozygous alleles out of 537358 | Absent | 6 heterozygous alleles out of 537358 | Absent |
| **Queen Square Genome database**  **(23 000 exomes)** | Absent | Absent | Absent | Absent | Absent |
| **Centogene database** | 1 heterozygous allele  Centogene AF ~5.6 x 10(-6) | 2 heterozygous alleles | Absent | Absent | Absent |
| **TOPMed** | 1 heterozygous allele | 1 heterozygous allele | Absent | Absent | Absent |
| **ClinVar** | Not reported | Not reported | Not reported | Not reported | Not reported |
| **CADD PHRED score** | 26.4 | 25.8 | 25 | 26 | 27.3 |
| **GERP** | 6.17 | 6.17 | 5.05 | 6.17 | 6.17 |
| **SIFT score** | 0.02  (Deleterious) | 0.00  (Deleterious) | 0.01  (Deleterious) | 0.00  (Deleterious) | 0.00  (Deleterious) |
| **PolyPhen score** | 0.964  (Probably damaging) | 0.946  (Probably damaging) | 1  (Probably damaging) | 0.999  (Probably damaging) | 0.994  (Probably damaging) |
| **PROVEAN** | -5.0  (Damaging) | -3.73  (Damaging) | -1.88  (Neutral) | -6.55  (Damaging) | -6.98  (Damaging) |
| **FATHMM PRED** | Tolerated | Tolerated | Tolerated | Tolerated | Damaging |
| **LRT PRED** | Deleterious | Deleterious | Deleterious | Deleterious | Deleterious |
| **MetaLR score** | 0.5429  (Damaging) | 0.5090  (Damaging) | 0.4605  (Tolerated) | 0.6008  (Damaging) | 0.5524  (Damaging) |
| **MutPred score** | 0.859 | 0.728 | 0.776 | 0.734 | 0.751 |
| **MutationAssessor score** | 3.42  (High functional impact) | 3.765  (High functional impact) | 2.845  (Medium functional impact) | 3.19  (Medium functional impact) | 3.38  (Medium functional impact) |
| **REVEL score** | 0.624 | 0.570 | 0.50 | 0.7039 | 0.7179 |
| **ACMG** | PS4, PP1 (moderate), PM2, PP3  Likely pathogenic | PS4, PP1 (moderate), PM2, PP3  Likely pathogenic | PM2, PP3, PP4  VUS | PM2, PP3  VUS | PM2, PP3  VUS |
